# Transcriptome analysis reveals the mechanism of internode development affecting maize stalk strength

**DOI:** 10.1186/s12870-022-03435-w

**Published:** 2022-01-24

**Authors:** Liuyong Xie, Daxing Wen, Chenglai Wu, Chunqing Zhang

**Affiliations:** State Key Laboratory of Crop Biology, Agronomy College, Shandong Agricultural University, Tai’an, Shandong Province 271018 P. R. China

**Keywords:** Maize, Lodging, Stalk strength, Rind, Cell wall

## Abstract

**Background:**

The stalk rind is one of the important factors affecting maize stalk strength that is closely related to stalk lodging. However, the mechanism of rind development in maize is still largely unknown.

**Results:**

In this study, we analyzed the mechanical, anatomical, and biochemical properties of the third basal internode in one maize non-stiff-stalk (NSS) line and two stiff-stalk (SS) lines. Compared with the NSS line, the two SS lines had a significantly higher rind penetrometer resistance, thicker rind, and higher dry matter, hemicellulose, cellulose, and lignin weights per unit length. RNA-seq analysis was used to compare transcriptomes of the third basal internode of the two SS lines and the NSS line at the ninth leaf and tasseling stages. Gene Ontology (GO) enrichment analysis revealed that genes involved in hydrolase activity (hydrolyzing O-glycosyl compounds) and cytoskeleton organization were significantly up-regulated in the two SS lines at the ninth leaf stage and that microtubule process-related genes were significantly up-regulated in the two SS lines at the tasseling stage. Moreover, the two SS lines had enhanced expression of cell wall metabolism-related genes at the tasseling stage.

**Conclusions:**

The synthesis of cell wall polysaccharides and the cytoskeleton might play important roles in internode development. Our results can be applied for screening lodging-resistant inbred lines and breeding lodging-resistant cultivars in maize.

**Supplementary Information:**

The online version contains supplementary material available at 10.1186/s12870-022-03435-w.

## Background

Stalk lodging affects not only maize yield but also mechanized harvesting efficiency. During lodging, the spatial distribution of leaves is disturbed, which alters photosynthetic efficiency. Stalk breakage may hinder sufficient transport of water and nutrients or lead to death of the entire plant. In addition, maize stalk lodging reduces ear position, which affects mechanized harvesting efficiency. Previous studies have shown that strong stalks significantly reduce stalk lodging [1, 2]. Therefore, studying mechanisms regulating stalk strength may provide new insights into stalk lodging resistance.

The maize stalk is composed of the pith and the rind, the latter mainly consisting of the epidermis and subepidermal lignified sclerenchyma cells [3]. Sclerenchyma cells have a thickened secondary cell wall that affects the strength and rigidity of plant tissues [4]. Therefore, rind thickness is closely related to rind penetrometer resistance (RPR) [5]. Optimization of cell wall synthesis and assembly can increase cell wall thickness, which is critical for improving stalk strength. However, the mechanism of sclerenchyma development in maize stalks is still unclear.

The primary cell wall, consisting of polysaccharides and smaller amounts of structural proteins, is synthesized in growing cells [6]. Once growth ceases, sclerenchyma cells deposit a thick lignified secondary wall containing more polysaccharides. Cellulose consisting of β-1,4-linked glucan chains is the main polysaccharide of plant cell walls [7]. In addition, many matrix polysaccharides, mainly pectin and hemicellulose, have been found in plant cell walls. Pectin polysaccharides include homogalacturonan, and rhamnogalacturonan I and II [8]. Hemicelluloses in plant cell walls include mannans, glucomannans, xylans, xyloglucans, and β-(1 → 3,1 → 4)-glucans [9]. Cellulose is synthesized by cellulose synthase (CesA) complexes (CSCs) embedded in the plasma membrane and is organized as microfibrils along the direction of microtubules. Disruption of cortical microtubules, which has revealed that membrane-localized CSCs are disorganized, does not affect the transport of CSCs from endomembranes to the plasma membrane or the velocity of CSCs at the plasma membrane [10]. Non-cellulosic polysaccharides are assembled in the Golgi apparatus and transported to the cell wall via vesicles [11]. When the cytoskeleton is damaged, vesicular transport and deposition at the cell walls are affected [12]. Improvement of maize stalk strength requires clarification of the stalk cell wall synthesis pathways. Although some studies on cell wall synthesis have been carried out, the internode development of maize inbred lines having different stalk strengths at different developmental stages remains poorly understood.

In this study, we analyzed the mechanical, anatomical, and biochemical properties of the third basal internode in one NSS line and two SS lines. We found that the two SS lines had a higher RPR than the NSS line. Further analysis revealed that the two SS lines had thicker rinds, rind and vascular bundle sheath cell walls, and more dry matter, hemicellulose, cellulose, and lignin per unit length than the NSS line. To explore genes and gene networks potentially playing important roles in regulating stalk strength in maize, we analyzed the transcriptomes of the third basal internode in the three inbred lines having different stalk strengths at the ninth leaf and tasseling stages. According to our results, genes involved in hydrolase activity (hydrolyzing O-glycosyl compounds) and cytoskeleton organization were significantly up-regulated in the two SS lines at the ninth leaf stage. Moreover, the two SS lines exhibited enhanced expression of cell wall metabolism-related genes at the tasseling stage. Taken together, our results provide new insights into the mechanism of internode development affecting maize stalk strength.

## Results

### Comparison of stalk strength-related traits among three maize inbred lines

**Fig. 1:**
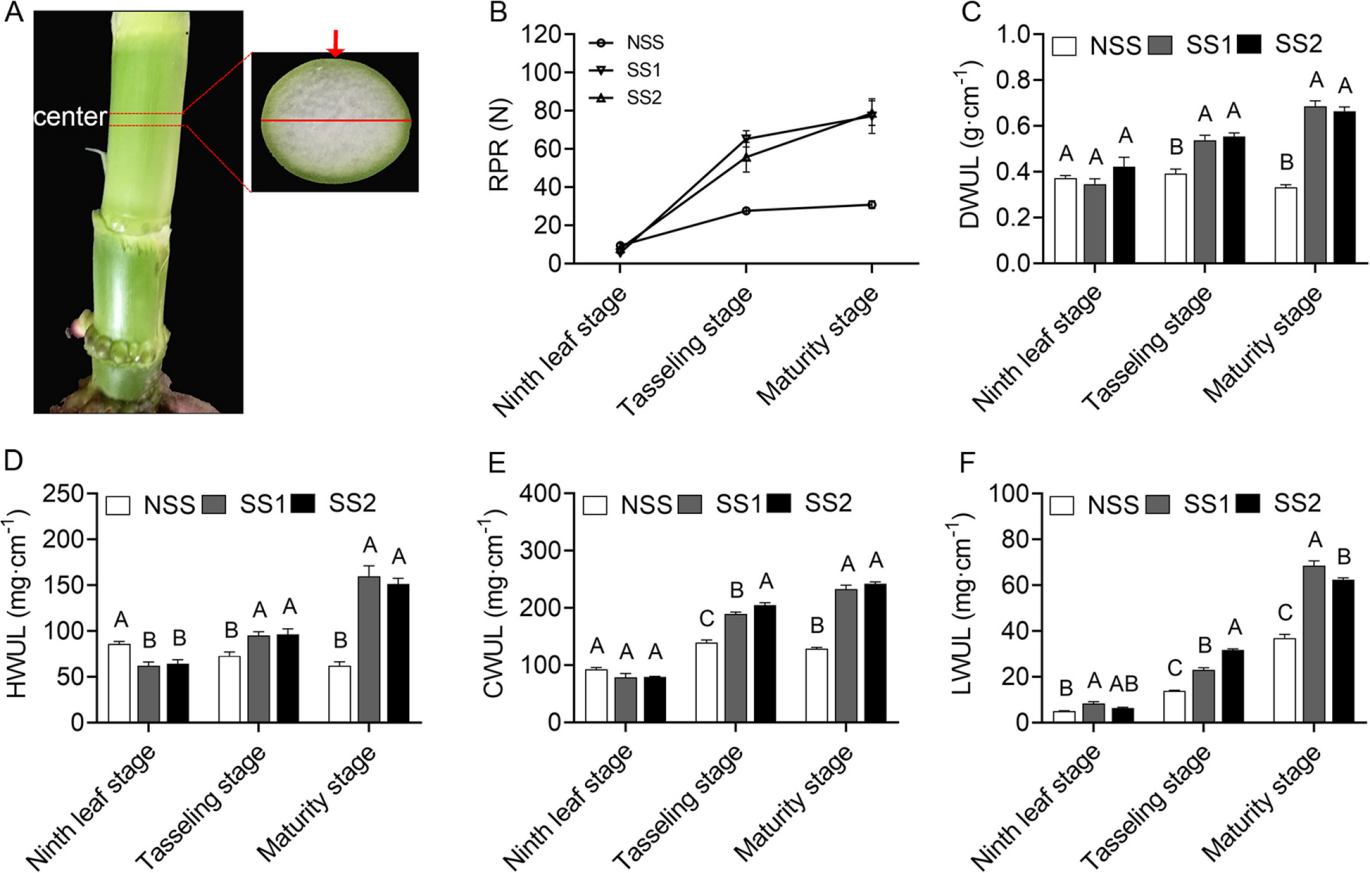
Rind penetrometer resistance (RPR) and stalk chemistry composition of two stiff-stalk (SS) lines and a non-stiff-stalk (NSS) line. **A** Stem region subjected to RPR measurement, paraffin sectioning, and RNA-seq sampling. The cross-sectional area of the probe was 1 mm^2^. Approximately 2 mm of the stem center was separated along the long axis of the cross section. The red arrow indicates the location of the RPR measurement, and the red line indicates the long axis. **B** RPR of the third basal internode. **C**–**F** Dry weight per unit length (DWUL), hemicellulose content per unit length (HWUL), cellulose content per unit length (CWUL), and lignin content per unit length (LWUL) of the third basal internode. Error bars represent standard deviation (*n* = 3). Different letters indicate significant differences at the same developmental stage (*p* < 0.01 by one-way ANOVA)

To identify differences in stalk strength, we analyzed RPR in one NSS and two SS inbred lines. No significant difference in RPR was detected between the two SS lines and the NSS line at the ninth leaf stage, but the two SS lines had markedly higher RPRs than the NSS line at the tasseling and maturity stages (Fig. 1A–B). Although SS2 was taller and had notably longer stem diameters and internode lengths than NSS, no significant difference was observed in the three traits between NSS and SS1 (Fig. S1). To investigate the effects of cell wall structural material contents on stalk strength, we measured the dry weight per unit length (DWUL), hemicellulose (HWUL), cellulose (CWUL), and lignin (LWUL) weights per unit length of the third basal internode (Fig. 1C–F). No significant difference was found in DWUL, CWUL, and LWUL between the two SS lines and the NSS line at the ninth leaf stage, but HWUL in the NSS line was higher than those in the two SS lines. At the tasseling and maturity stages, the two SS lines had higher DWUL and cell wall structural material contents (HWUL, CWUL, and LWUL) than the NSS line. The DWUL and cell wall structural material contents (HWUL, CWUL, and LWUL) of the two SS lines gradually increased from the ninth leaf stage to the maturity stage. In the NSS line, LWUL exhibited a similar trend as in the two SS lines, whereas HWUL decreased over time. Moreover, DWUL and CWUL in the NSS line first increased and then decreased from the ninth leaf stage to the maturity stage.

**Fig. 2:**
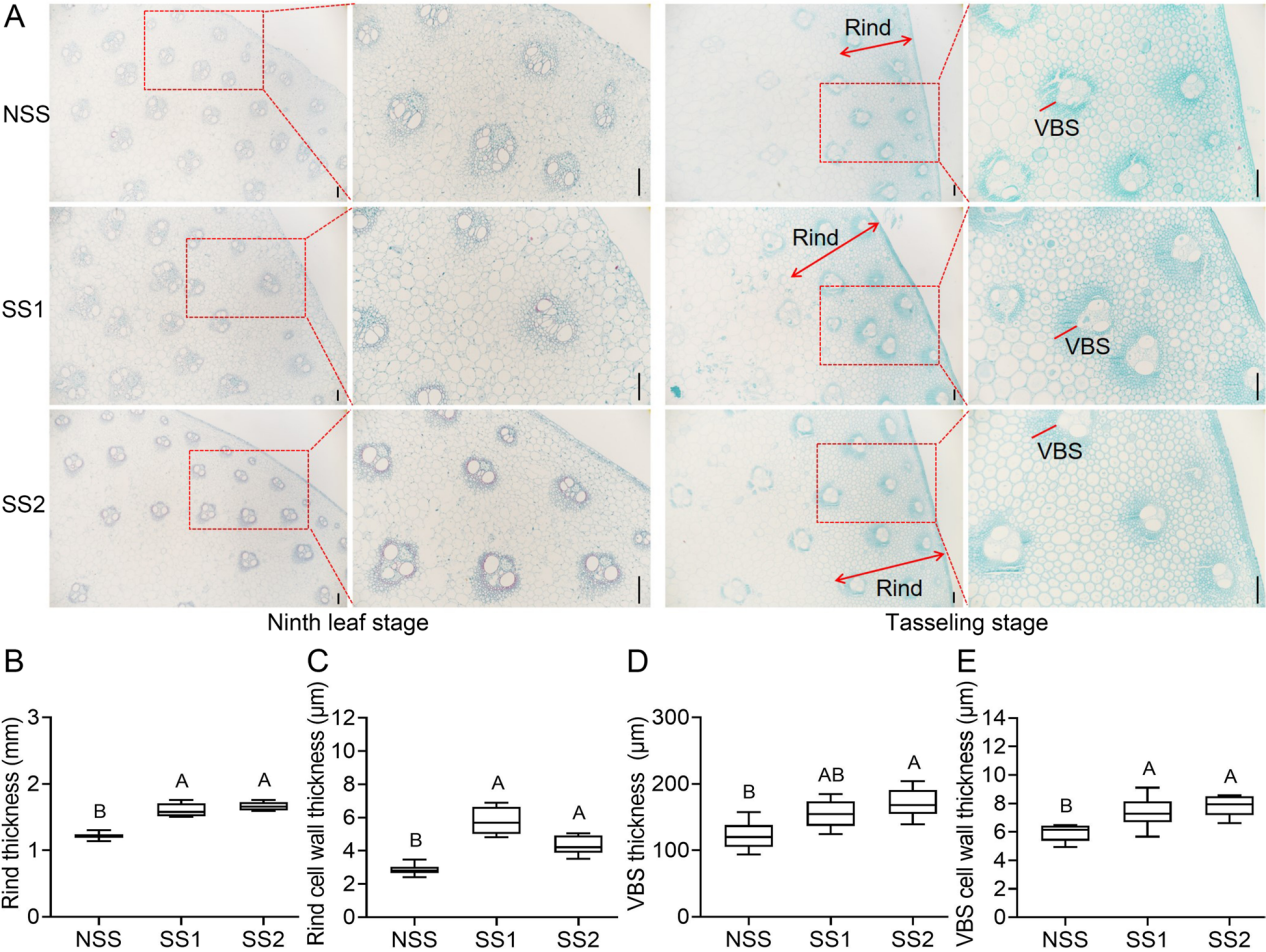
Microstructure of the third basal internode of two stiff-stalk (SS) lines and a non-stiff-stalk (NSS) line. **A** Images of the cross section of the center of the third basal internode. The double-ended arrow indicates the rind width. The red line indicates vascular bundle sheath (VBS) thickness. Scale bar = 200 μm. **B**–**E** Rind, rind cell wall, VBS, and VBS cell wall thicknesses at the tasseling stage. Error bars represent the standard deviation (*n* = 10 for **B** and **D**; *n* = 20 for **C** and **E**). Different letters indicate significant differences at the same developmental stage (*p* < 0.01 by one-way ANOVA)

To investigate differences in the stalk microstructure of the NSS line and the two SS lines, we observed cross sections of the third basal internode at the ninth leaf and tasseling stages (Fig. 2A). No significant difference in stalk microstructure was detected between the NSS line and the two SS lines at the ninth leaf stage. At the tasseling stage, no significant difference was observed in the pith of the stem between the NSS line and the two SS lines. However, the two SS lines had notably thicker rinds than the NSS line (Fig. 2A). We subsequently measured stalk rind, rind cell wall, vascular bundle sheath (VBS), and VBS cell wall thicknesses at the tasseling stage (Fig. 2B–E). The rind, VBS, rind cell, and VBS cell wall thicknesses of the two SS lines were markedly larger than those of the NSS line at the tasseling stage. Although there was no significant difference in VBS thickness between the SS1 line and NSS line, the SS2 line had a notably larger VBS thickness than the NSS line. At the ninth leaf stage, the early stage of stalk structure formation, rind tissue development was incomplete. At the tasseling stage, the two SS lines had significantly thicker rind and thick-walled cells than the NSS line. Therefore, the differences in stalk strength between the two SS lines and the NSS line were associated with rind and cell wall thicknesses.

### Comparison of the expression levels of genes related to cell wall metabolism between the two SS lines and the NSS line

**Fig. 3:**
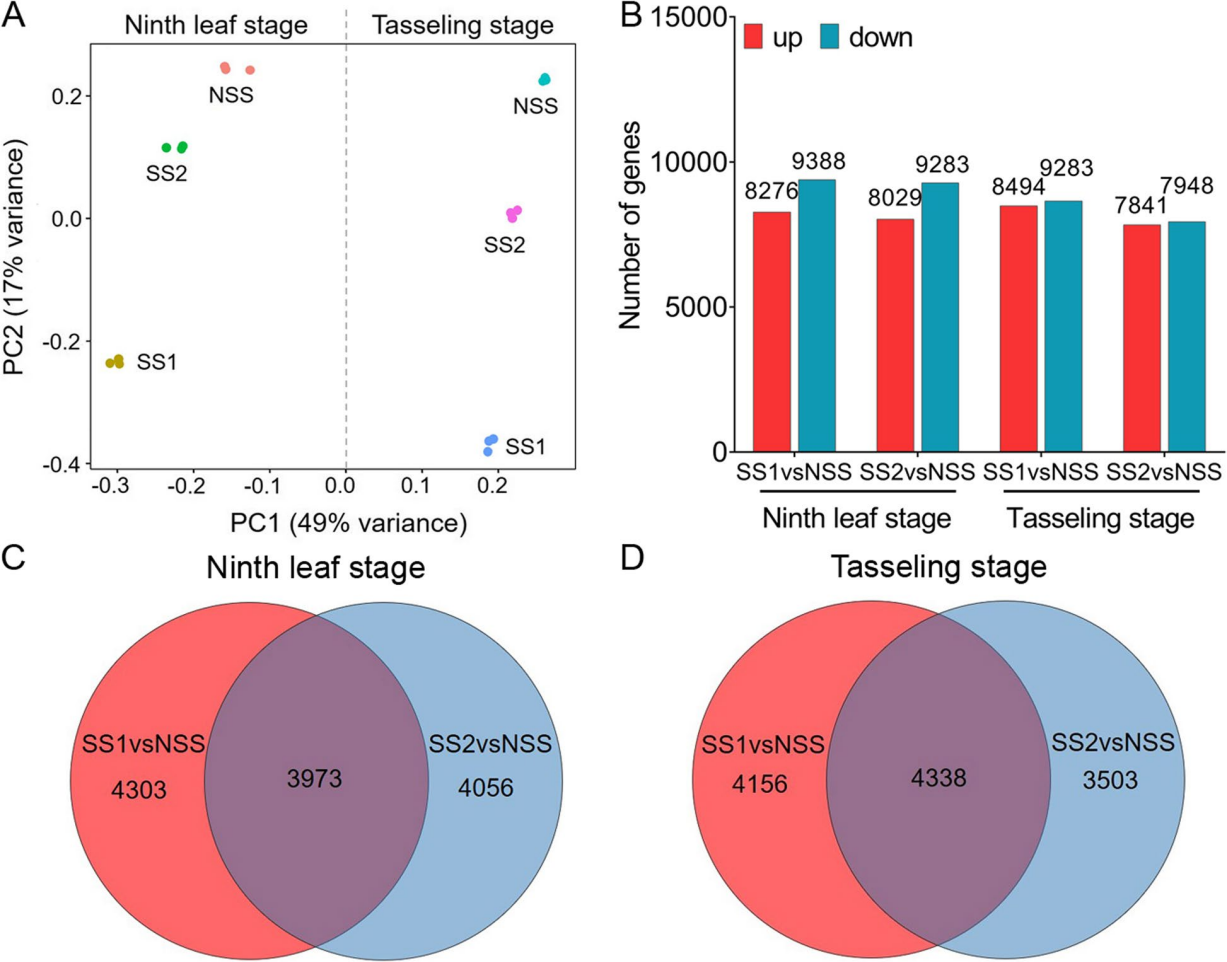
Transcriptome analysis in two stiff-stalk (SS) lines and a non-stiff-stalk (NSS) line. **A** Principal component analysis (PCA) of all six samples. **B** Number of up-regulated (up) and down-regulated (down) DEGs between the two SS lines and the NSS line at the ninth leaf and tasseling stages. Venn diagram of up-regulated DEGs in SS1vsNSS and SS2vsNSS at the ninth leaf stage (**C**) and the tasseling stage (**D**). SS1vsNSS: stiff-stalk-line HB08F1 compared with non-stiff-stalk-line SJ20104; SS2vsNSS: stiff-stalk-line A801 compared with non-stiff-stalk-line SJ20104

To explore the mechanism by which internode development affects maize stalk strength, we analyzed the transcriptomes of the third basal internode at two stages (ninth leaf stage and tasseling stage) by RNA-seq. After removing low-quality regions and adapter sequences, approximately 51.16 to 76.11 million clean reads remained (Table S1). Approximately 46.12 to 68.81 million clean reads were mapped to the maize B73 reference genome (v4.0). In each library, 80.09 to 88.59% of clean reads were uniquely mapped reads (Table S1). In a principal component analysis (PCA), biological replicates of the same treatment clustered together, which implied that the transcriptome datasets were satisfactory (Fig. 3A). The first principal component was able to discriminate between samples at the ninth leaf stage and the tasseling stage, which indicates that the transcriptome datasets from these two stages were markedly different.

Genes with fragments per kilobase of transcript sequence per million base pairs sequenced (FPKM) ≥ 1 were considered to be expressed genes. According to this criterion, 21,819, 21,663, and 22,023 genes were expressed in NSS, SS1, and SS2, respectively, at the ninth leaf stage. At the tasseling stage, there were 20,882, 21,241, and 20,984 expressed genes in NSS, SS1, and SS2, respectively. After filtering out genes with low expression (FPKM < 1 in all three inbred lines), 25,751 and 25,088 genes were expressed at the ninth leaf and tasseling stages, respectively. We then used the R package DESeq to identify differentially expressed genes (DEGs). Compared with the NSS line, approximately 8000 DEGs each were significantly up-regulated and significantly down-regulated in the two SS lines (Fig. 3B). To reduce the effects of material background on the results, we applied a Venn diagram to screen common DEGs in SS1vsNSS and SS2vsNSS (Fig. 3C–D). With regard to up-regulated DEGs, we identified 3973 and 4338 common DEGs at the ninth leaf and tasseling stages, respectively. To further analyze the function of these common DEGs, we performed a Gene Ontology (GO) enrichment analysis (*p* < 0.05). In a subsequent transcriptome analysis, we focused on DEGs and GO terms involved in cell wall metabolism given the differences in thick-walled cells between the two SS lines and the NSS line.

### Involvement of hydrolyzing O-glycosyl compounds and cytoskeleton organization-related genes in internode development at the ninth leaf stage

**Fig. 4:**
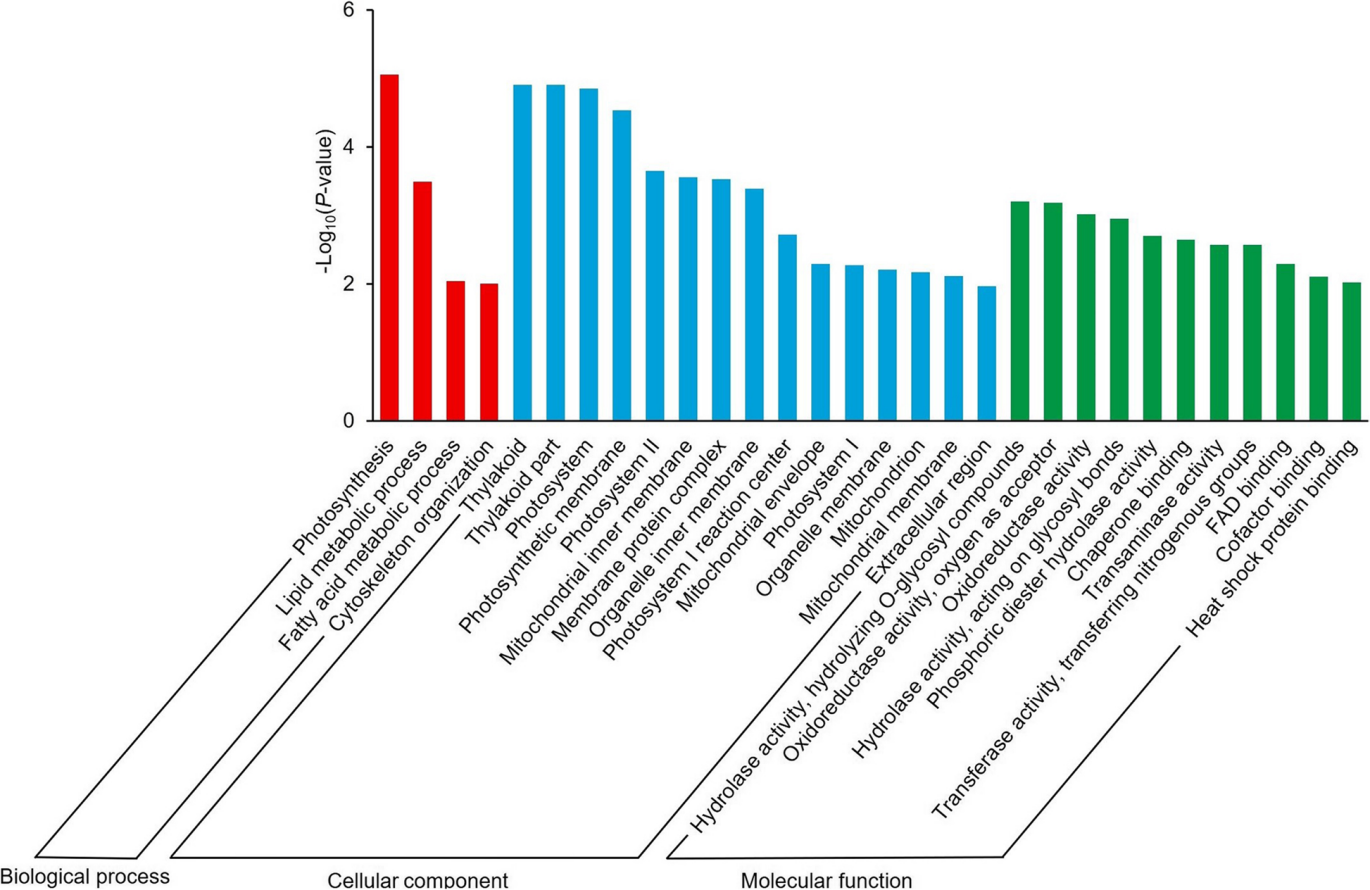
Top 30 significantly enriched Gene Ontology (GO) terms (*p* < 0.05) in DEGs commonly up-regulated in SS1vsNSS and SS2vsNSS at the ninth leaf stage. SS1vsNSS: stiff-stalk-line HB08F1 compared with non-stiff-stalk-line SJ20104; SS2vsNSS: stiff-stalk-line A801 compared with non-stiff-stalk-line SJ20104

There were 3973 common up-regulated DEGs in SS1vsNSS and SS2vsNSS at the ninth leaf stage (Fig. 3C). GO enrichment analysis of these common up-regulated DEGs revealed that the most significantly enriched GO terms were photosynthesis (GO: 0015979, *p* = 8.79 × 10^− 6^) in the biological process group, thylakoid (GO: 0009579, *p* = 1.24 × 10^− 5^) in the cellular component group, and hydrolase activity (hydrolyzing O-glycosyl compounds) (GO: 0004553, *p* = 6.19 × 10^− 4^) in the molecular function group (Fig. 4). Among them, 64 DEGs were involved in hydrolase activity (hydrolyzing O-glycosyl compounds) (GO: 0004553, *p* = 6.19 × 10^− 4^, Table S2). Many of these 64 DEGs were involved in cell wall metabolism. The largest group comprised glycosyl hydrolase family genes (36 DEGs). In addition, genes related to xyloglucan endo-transglycosylase (XET, *Zm00001d002410*, *Zm00001d014613*, *Zm00001d014617*, *Zm00001d017699*, and *Zm00001d029814*), beta-glucosidase 2 (*Zm00001d005431* and *Zm00001d048988*), and cellulase (*Zm00001d010039*) were markedly up-regulated in SS1vsNSS and SS2vsNSS. Moreover, 11 genes implicated in cytoskeleton organization (GO: 0007010, *p* = 1.00 × 10^− 2^) (Table S3) were notably up-regulated in the SS lines. Therefore, the two SS lines had significantly higher expression levels of cell wall metabolism-related genes than the NSS line, which suggests that the difference in cell wall metabolism between the NSS line and the two SS lines had already occurred at the ninth leaf stage.

### Cell wall metabolism-related genes are implicated in the internode development of inbred lines with different stalk strengths at the tasseling stage

**Fig. 5:**
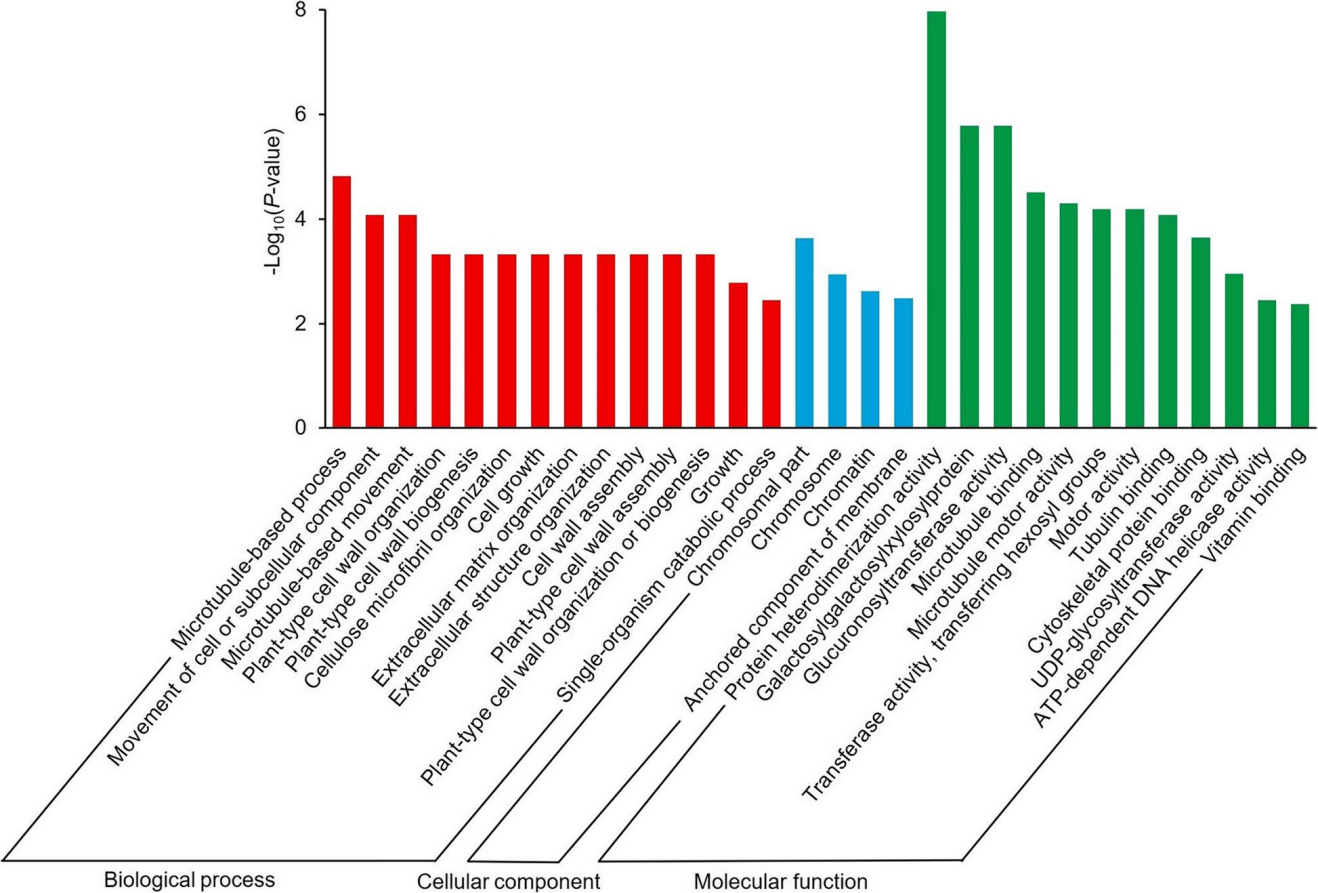
Top 30 significantly enriched Gene Ontology (GO) terms (*p* < 0.05) in DEGs commonly up-regulated in SS1vsNSS and SS2vsNSS at the tasseling stage. SS1vsNSS: stiff-stalk-line HB08F1 compared with non-stiff-stalk-line SJ20104; SS2vsNSS: stiff-stalk-line A801 compared with non-stiff-stalk-line SJ20104

At the tasseling stage, 4338 DEGs were commonly up-regulated in SS1vsNSS and SS2vsNSS (Fig. 3D). For these commonly up-regulated DEGs, the most remarkably significantly enriched GO terms were the microtubule-based process (GO: 0007017, *p* = 1.52 × 10^− 5^) in the biological process group, chromosomal part (GO: 0044427, *p* = 2.36 × 10^− 4^) in the cellular component group, and protein heterodimerization activity (GO: 0046982, *p* = 1.06 × 10^− 8^) in the molecular function group (Fig. 5). In regard to microtubule-based processes (GO: 0007017, *p* = 1.52 × 10^− 5^), more than half (20/36) of the DEGs encoded proteins that were predicted to have a kinesin motor domain (Table S4). In *Arabidopsis thaliana*, kinesin-4-based transport of non-cellulose substances along cortical microtubules is associated with cell wall mechanics [13]. Therefore, we speculate that DEGs involved in microtubule-based processes might affect the supply of synthetic substances in the cell wall. Moreover, many GO terms involved in cell wall metabolism were significantly enriched in the biological process group, such as plant-type cell wall biogenesis (GO: 0009832, *p* = 4.76 × 10^− 4^), cellulose microfibril organization (GO: 0010215, *p* = 4.76 × 10^− 4^), and cell wall assembly (GO: 0070726, *p* = 4.76 × 10^− 4^). Many more cell wall metabolism-related GO terms were enriched at the tasseling stage than at the ninth leaf stage, which indicates that differences in cell wall metabolism between the NSS line and the two SS lines became more pronounced at the tasseling stage.

**Fig. 6:**
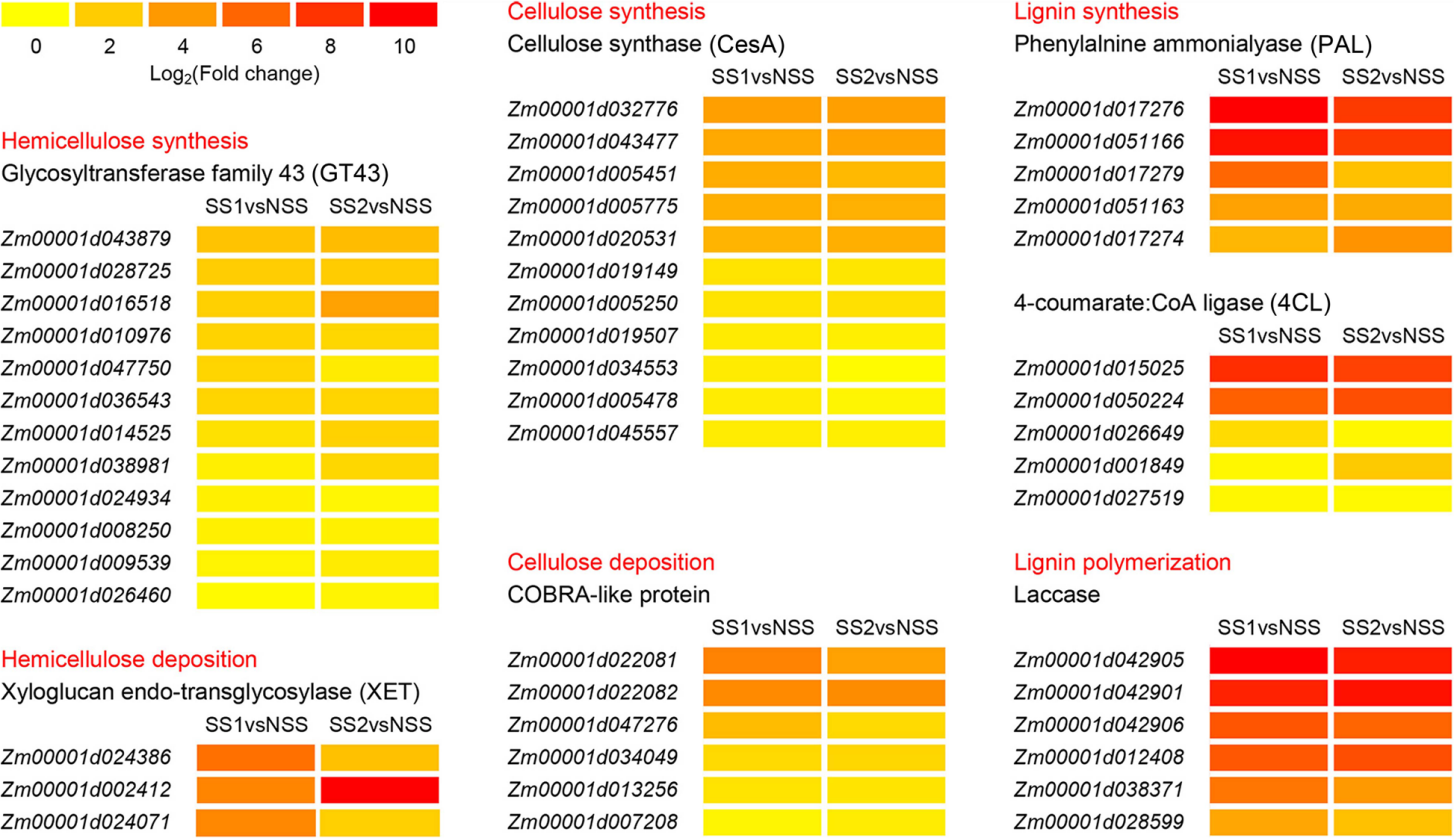
Heatmap of genes related to cell wall synthesis at the tasseling stage. SS1vsNSS: stiff-stalk-line HB08F1 compared with non-stiff-stalk-line SJ20104; SS2vsNSS: stiff-stalk-line A801 compared with non-stiff-stalk-line SJ20104

Many genes related to the synthesis of cell wall components were significantly enriched in transferase activity (transferring hexosyl groups) (GO: 0016758, *p* = 6.46 × 10^− 5^). Most of the transferase activity related genes were UDP-glucoronosyl and UDP-glucosyl transferase genes (33/85, Table S5). The glycosyltransferase (GT) family GT43 is involved in the biosynthesis of xylan, which is a hemicellulose [14, 15]. Compared with the NSS line, 12 GT43 family genes were up-regulated in the two SS lines at the tasseling stage (Fig. 6). In addition, although the two SS lines had higher CWUL than the NSS line at the tasseling stage, 11 cellulose synthase genes were still significantly up-regulated in the two SS lines compared with the NSS line, thereby resulting in further enlargement of the difference in CWUL between the NSS line and the two SS lines at the maturity stage (Figs. 1E and 6 and Table S5). Lignin synthesis-related GO terms were not notably enriched at the tasseling stage, but the two SS lines already had significantly higher LWUL than the NSS line. Therefore, we also analyzed the expression pattern of lignin synthesis-related genes, including phenylalanine ammonialyase (*PAL*), 4-coumarate:CoA ligase (*4CL*), and *laccase*. *PAL* and *4CL* are involved in lignin synthesis [16, 17], and *laccase* genes involved in lignin polymerization [18] have been reported in *Arabidopsis* and maize. The results showed that five *PAL* genes, five *4CL* genes, and six *laccase* genes were up-regulated in both SS lines (Fig. 6 and Table S6). These genes might play important roles in the biosynthesis of cell wall components and enhancing stalk strength.

Hemicellulose and cellulose deposition form the secondary cell wall network, which affects cell wall mechanics [19, 20]. Xyloglucan endotransglucosylase (XET) cuts and rejoins hemicellulose chains to promote plant cell wall assembly and growth regulation [21]. Previous studies have identified a role for XET in secondary cell wall deposition [22]. Three *XET* (*Zm00001d024386*, *Zm00001d024071*, and *Zm00001d002412*) genes were up-regulated in the two SS lines at the tasseling stage (Fig. 6 and Table S5). Mutation in the gene encoding a COBRA-like protein in *Arabidopsis* affects cellulose deposition [23]. We found that six DEGs were enriched in plant-type cell wall organization (GO: 0009664, *p* = 4.76 × 10^− 4^). All six DEGs were predicted to encode COBRA-like proteins and were up-regulated in the two SS lines at the tasseling stage (Fig. 6 and Table S7). In rice, the deletion of a COBRA-like protein gene causes a reduction in cellulose content, resulting in decreases in secondary cell wall thickness and mechanical strength [24]. In particular, mutants of *brittle stalk2* (*Zm00001d047276*) display brittle organs (including stems) [25]. Therefore, many genes involved in cell wall metabolism were up-regulated in the two SS lines compared with the NSS line, which is consistent with the observed trends in cell wall structural material contents.

### GO term enrichment analysis of down-regulated DEGs in SS1vsNSS and SS2vsNSS

At the ninth leaf stage, 5315 common DEGs were down-regulated in SS1vsNSS and SS2vsNSS (Fig. S2A). Among these common down-regulated DEGs, GO term enrichment analysis showed that the most significantly enriched GO terms were sulfate transport (GO: 0008272, *p* = 5.13 × 10^− 4^) and sulfur compound transport (GO: 0072348, *p* = 5.13 × 10^− 4^) in the biological process group and sulfate transmembrane transporter activity (GO: 0015116, *p* = 4.69 × 10^− 4^) in the molecular function group (Fig. S3). There was no significant GO term that might be involved in stalk strength. Venn diagram analysis revealed that 5138 common DEGs were down-regulated in SS1vsNSS and SS2vsNSS at the tasseling stage (Fig. S2B). The most significantly enriched GO terms related to these common down-regulated DEGs were photosynthesis (GO: 0015979, *p* = 9.65 × 10^− 11^) under biological process, the thylakoid (GO: 0009579, *p* = 3.18 × 10^− 12^) and thylakoid part (GO: 0044436, *p* = 3.18 × 10^− 12^) under cellular components, and iron-sulfur cluster binding (GO: 0051536, *p* = 1.80 × 10^− 6^) and metal cluster binding (GO: 0051536, *p* = 1.80 × 10^− 6^) under molecular function (Fig. S4). GO terms that might be involved in stalk strength were also not found at the tasseling stage. Therefore, up-regulated DEGs between the two SS lines and the NSS line might play more important roles in regulating stalk strength than down-regulated DEGs.

### Identification of differentially expressed transcription factors

**Fig. 7:**
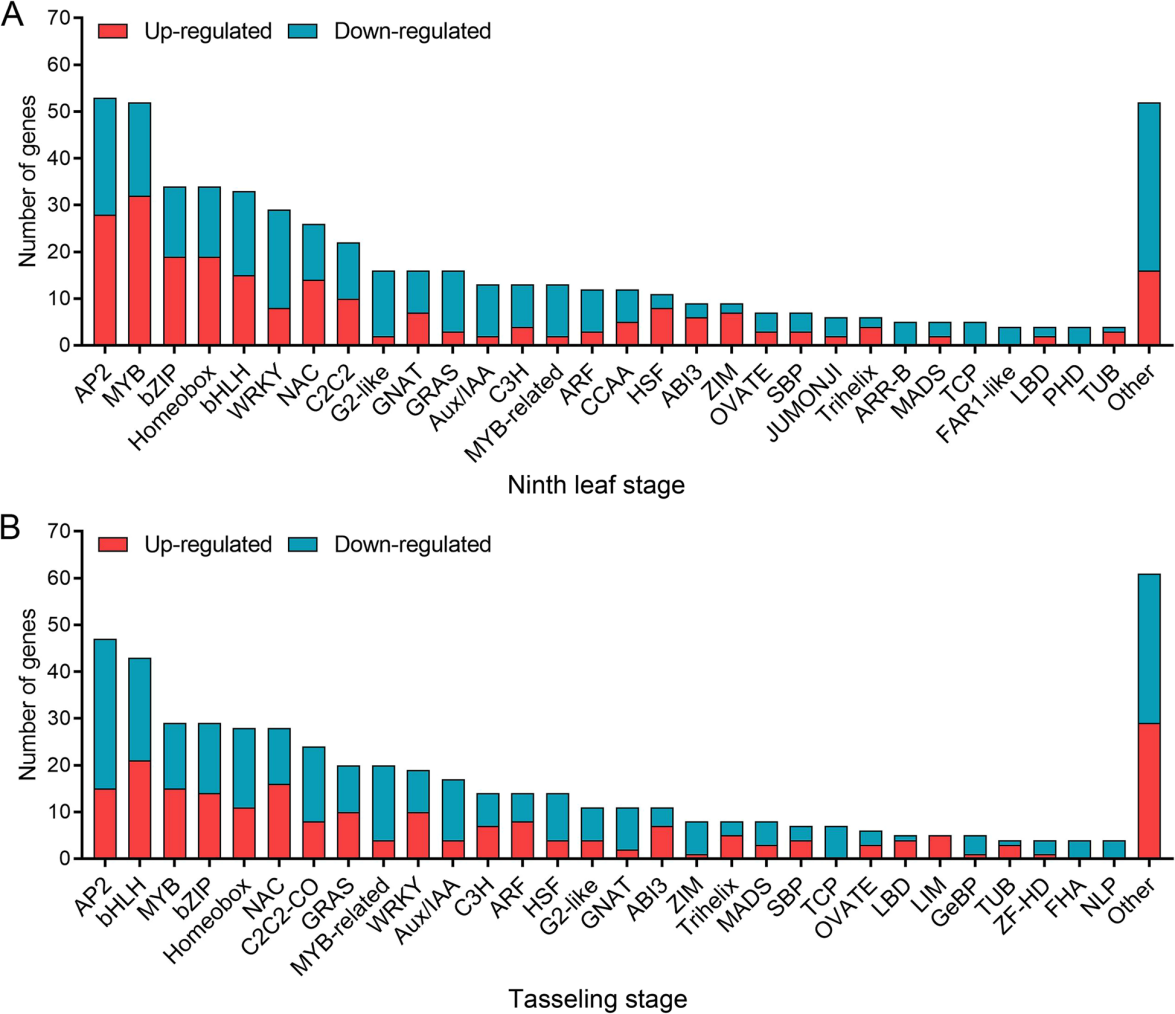
Differentially expressed TFs common in SS1vsNSS and SS2vsNSS. **A** Differentially expressed TFs common in SS1vsNSS and SS2vsNSS at the ninth leaf stage. **B** Differentially expressed TFs common in SS1vsNSS and SS2vsNSS at the tasseling stage. SS1vsNSS: stiff-stalk-line HB08F1 compared with non-stiff-stalk-line SJ20104; SS2vsNSS: stiff-stalk-line A801 compared with non-stiff-stalk-line SJ20104

Transcription factors (TFs) play important roles in cell wall metabolism [20]. To explore which TFs might be involved in internode development, we identified common differentially expressed TFs between SS and NSS lines by annotation in the PlantTFDB (http://planttfdb.gao-lab.org/index.php?sp=Zma) database. There were 514 differentially expressed TFs assigned to 45 families at the ninth leaf stage. The top 10 families by TF number were AP2/ERF, MYB, bHLH, bZIP, WRKY, NAC, C2H2, HD-ZIP, G2-like, and GRAS (Fig. 7A, Table S8). At the tasseling stage, 521 differentially expressed TFs were assigned to 47 families. The top 10 families by TF number were bHLH, AP2/ERF, bZIP, MYB, NAC, C2H2, GRAS, WRKY, HD-ZIP, and MYB_related (Fig. 7B, Table S8). AP2/ERF family TFs can regulate secondary cell wall formation [26]. In the present study, the AP2/ERF, bHLH, MYB, and bZIP families had notably more DEGs than the other families at both the ninth leaf and tasseling stages. The MYB and NAC families play important roles in the network of TFs that regulate secondary wall synthesis [27]. At the tasseling stage, NAC families also had a large number of differentially expressed TFs. These differentially expressed TFs might participate the in regulation of maize stalk strength, which needs validation in further study.

### Validation of RNA-seq data by qRT-PCR

**Fig. 8:**
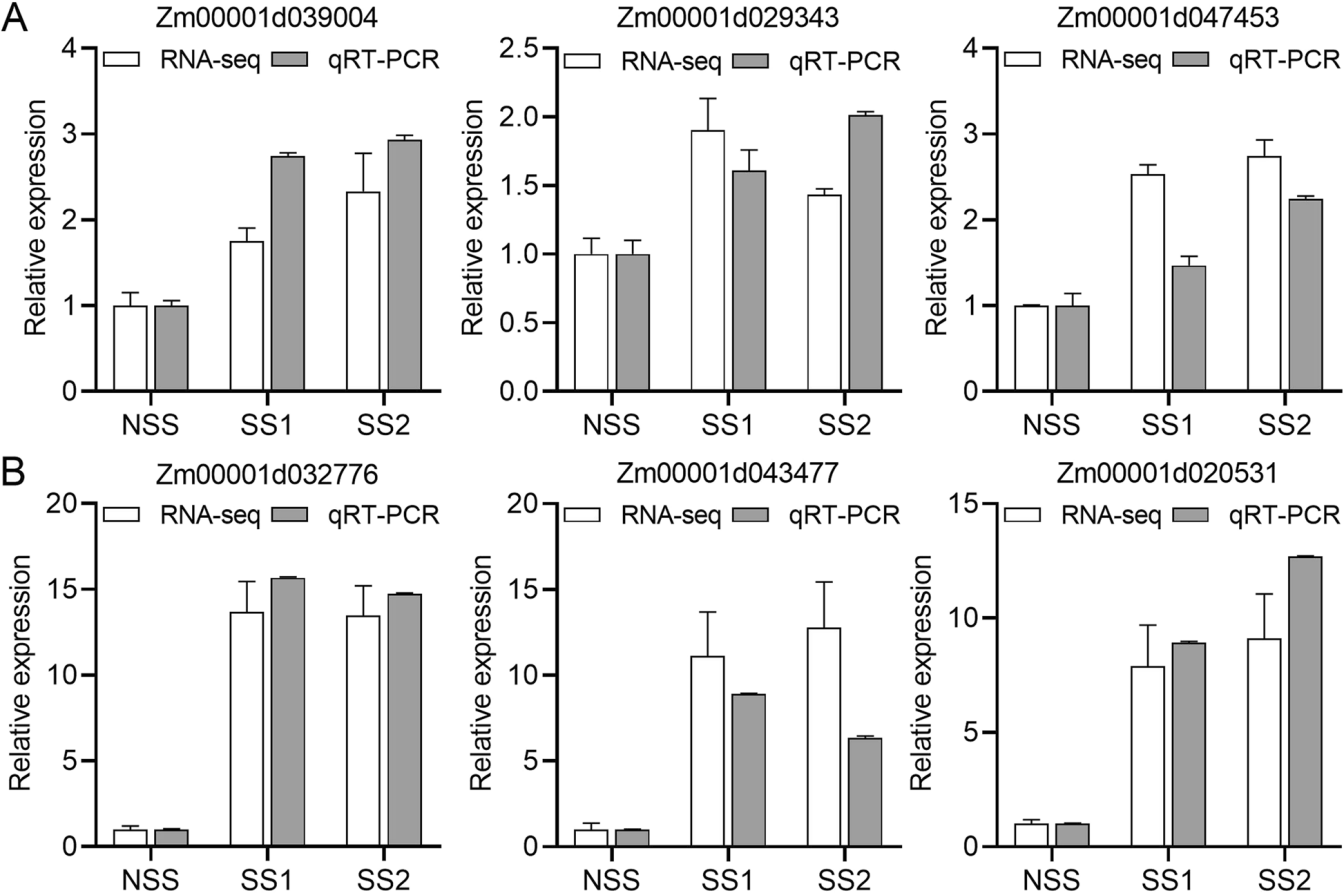
Validation of differentially expressed genes (DEGs) by qRT-PCR. **A** Three DEGs were up-regulated between the two SS lines and the NSS line at the ninth leaf stage. **B** Three DEGs were up-regulated between the two SS lines and the NSS line at the tasseling stage. The relative expression level of each gene was expressed as the fold change in RNA-seq data (white bar) and qRT-PCR data (gray bar). The maize *actin* gene was used as an internal control for normalization of gene expression. Error bars represent the standard deviation (*n* = 3)

To validate the DEGs identified by RNA-seq, we performed qRT-PCR assays using independently collected samples identical to those used for RNA-seq analysis at the same developmental stage. Among the six DEGs selected for validation, three DEGs were up-regulated between the two SS lines and the NSS line at the ninth leaf and tasseling stages (Fig. 8). *Zm00001d039004* encodes a NAC-transcription factor that shares high protein sequence homology with the secondary wall-associated NAC domain 2 (SND2) protein in rice [28]. *Zm00001d029343* was predicted to encode an F-actin capping protein. The *Zm00001d047453* encoded protein was predicted to have a villin headpiece domain. At the tasseling stage, we selected three cellulose synthase genes: *Zm00001d032776* (*CesA10*), *Zm00001d043477* (*CesA11*), and *Zm00001d020531* (*CesA12*). The expression patterns of all six DEGs in the qRT-PCR assays were similar to those in the RNA-seq data, thus indicating the high reliability of the RNA-seq data.

## Discussion

Maize stalks are composed of rind and pith. The rind is made up of hard and dense tissues, whereas the central pith of the stem is a much softer, foam-like tissue [29]. Previous research has noted the importance of rind thickness in maize stalk strength [2]. A recent study found that the cell walls of sclerenchyma cells of lodging-resistant lines were thicker than those of lodging-sensitive lines [30]. Similar results were observed in the present study. We found significant differences in rind thickness and cell wall thickness between the two SS lines and the NSS line (Fig. 2). Moreover, the two SS lines with stronger stalks had more hemicellulose, cellulose, and lignin (Fig. 1D–F). According to these results, the structural polysaccharides and lignins of cell walls are related to the mechanical strength of the stalk, similar to the findings of previous studies [31]. Therefore, the thickness of the rind and cell wall might play important roles in maize stalk strength.

The plant cell wall is a complex matrix mainly consisting of polysaccharides, proteins, and lignins [20]. In particular, the changes in polysaccharides (cellulose, hemicellulose, and pectin) affect the cell wall mechanical strength and the viscoelastic properties of tissue [32]. Cell wall polysaccharides (hemicellulose and pectin) are synthesized by glycosyltransferases (GTs) in the Golgi apparatus and secreted into the cell wall by secretory vesicles [33]. Genes belonging to glycosyltransferase (GT) families were enriched in the transferase activity (transferring hexosyl) group (GO: 0016758, *p* = 6.46 × 10^− 5^) at the tasseling stage (Table S5). In the present study, a large number of UDP-glucosyl transferase genes (33/85) were enriched in the transferase activity (transferring hexosyl) group (GO: 0016758, *p* = 6.46 × 10^− 5^) at the tasseling stage. UDP-glucosyl transferases mediate the transfer of glycosyl residues from activated nucleotide sugars (possibly UDP-glucose, UDP-galactose, UDP-glucuronic acid, or UDP-xylose) to acceptor molecules [34]. UDP glucose is a major substrate for the synthesis of cell wall polysaccharides, including cellulose, callose, xyloglucan, and glucomannan [35]. Xyloglucan and xylan are the two major hemicellulose polysaccharides in the cell walls of plants [14]. In *Arabidopsis*, the irregular xylem 9/−like (*IRX9/IRX9-L*) and *IRX14/IRX14-L* genes encode members of GT43, and the destruction of these proteins results in a decrease in xylan content [36]. In the present study, 12 GT43 genes, including the homologous genes of *IRX9* (*Zm00001d010976*) and *IRX14* (*Zm00001d036543*), were up-regulated in the two SS lines compared with the NSS line at the tasseling stage (Fig. 6 and Table S5). In maize, *CesA10*, *CesA11* and *CesA12* are cellulose synthases [37]. Compared with the NSS line, *CesA10* (*Zm00001d032776*), *CesA11* (*Zm00001d043477*) and *CesA12* (*Zm00001d020531*) were all up-regulated in the two SS lines at the tasseling stage (Fig. 6 and Table S5). At the tasseling stage, maize inbred lines with different stalk strengths exhibited marked differences in cell wall synthesis. Compared with the NSS line, the two SS lines had higher cell wall polysaccharide contents (Fig. 1), which suggests that the two SS lines require more cell wall polysaccharides.

When cell wall polysaccharides produced in the Golgi apparatus are secreted into cell walls, multiple exoplasmic enzymes modify the structure of non-cellulosic polysaccharides, such as glycosyl hydrolases (GHs) [38, 39]. Xyloglucan endotransglucosylase/hydrolase (XTH), belonging to the GH16 family, plays a role in the plant cell wall structure and function [40]. XTH proteins have the activity of xyloglucan endotransglycosylase (XET) and xyloglucan endohydrolase, and act on xyloglucan attached to cellulose microfibers [41, 42]. In previous studies on XET activity in poplar, XET was found to play a role in secondary cell wall deposition [22]. In the present study, three XETs were up-regulated in two SS lines at the tasseling stage, which might affect hemicellulose deposition in the secondary cell wall (Fig. 6 and Table S5).

Mutation of the *Arabidopsis* COBRA-like 10 gene leads to the destruction of cellulose microfibril deposition in pollen tubes [23]. In rice, mutation of the COBRA-like protein gene *brittle culm1* leads to a decrease in cellulose content and secondary cell wall thickness [24]. In the present study, six genes were predicted to encode COBRA-like proteins and were up-regulated in two SS lines at the tasseling stage (Fig. 6 and Table S7). Maize *brittle stalk 2* encodes a COBRA-like protein similar to the rice brittle culm1 protein. Mutation in *brittle stalk 2* affects cellulose deposition in the secondary cell wall and stalk strength [25, 43]. Here, *brittle stalk 2* (*Zm00001d022081*) was up-regulated in the two SS lines at the tasseling stage (Fig. 6 and Table S7). Therefore, there might be a marked difference in cellulose deposition between the two SS lines and the NSS line.

Lignin, the second most abundant cell wall structural material after cellulose in the secondary cell wall, is very important for stem strength [44]. Previous studies found significantly reduced lignin content in *Arabidopsis PAL1* and *PAL2* double mutants [16]. In the present study, five *Arabidopsis PAL1* and *PAL2* homologous genes, *ZmPAL1* (*Zm00001d017274*), *ZmPAL4* (*Zm00001d051166*), *ZmPAL5* (*Zm00001d051163*), *ZmPAL7* (*Zm00001d017279*), and *ZmPAL8* (*Zm00001d017276*), were up-regulated in the two SS lines at the tasseling stage (Fig. 6 and Table S6). A recent study found that the *Zm4CL1* mutation mainly affected the biosynthesis of G lignin and the accumulation of soluble feruloyl derivatives in maize lignified tissues [17]. Although there was no significant difference in *Zm4CL1* expression in our RNA-seq data, we found that another five *4CL* genes were up-regulated in the two SS lines at the tasseling stage (Fig. 6 and Table S6). Laccase is necessary for lignin polymerization during vascular development in *Arabidopsis* [18]. Our results showed that six *laccase* genes were up-regulated at the tasseling stage (Fig. 6 and Table S6). Under nitrogen stress, ZmmiR528 affected the lignin content and RPR of maize stems by regulating the expression of *ZmLACCASE3* and *ZmLACCASE5* [45]. Here, *ZmLACCASE5* (*Zm00001d042901*) was up-regulated at the tasseling stage (Fig. 6 and Table S6). Taken together, enhanced expression of genes involved in cell wall synthesis and metabolism might increase cell wall structural material contents and cell wall thickness in the two SS lines, thereby improving stalk strength.

The cell wall structure is not only affected by the synthesis and modification of cell wall polysaccharides but is also regulated and organized by the dynamic cytoskeleton and multiple transport pathways of cell wall polymers [46]. The cytoskeleton is composed of microtubules and actin filaments (F-actin). Actin filament polymerization and elongation are related to vesicle mobility [47]. Microtubules are involved in the transport of vesicles containing cell wall components [48]. Cytoskeleton-associated proteins affect cell wall mechanics and cell wall material deposition [13, 49].

In this study, genes related to cytoskeleton organization (Table S3) and microtubule-based processes (Table S4) were enriched at the ninth leaf and tasseling stages, respectively. Actin-related protein 2/3 (Arp2/3) complex and F-actin capping protein-related genes were up-regulated at the ninth leaf stage in the two SS lines compared with the NSS line. The Arp2/3 complex is a type of actin filament nucleation factor that extends a new actin filament with 70° branch angles from one side of existing actin filaments [50]. The F-actin capping protein is a heterodimer composed of α and β subunits that binds to the F-actin ends to limit the addition or loss of G-actin, thereby stabilizing the actin filament cytoskeleton [51]. Therefore, the Arp2/3 complex and F-actin capping proteins may affect the synthesis and nucleation of actin filaments and subsequently influence the transportation of cell wall polysaccharides during cell wall synthesis. In this study, capping protein subunits α (*Zm00001d007146*, log2FC = 0.44–0.54) and β (*Zm00001d029343*, log2FC = 0.48–0.89) were up-regulated in the two SS lines at the ninth leaf stage (Table S3). In addition, a large number of kinesin-related genes (20/36) were enriched in the GO term of the microtubule-based process (GO: 0007017, *p* = 1.52 × 10^− 5^). Kinesin-1 is a processive motor that uses ATP energy to transport cellular cargoes from the Golgi apparatus to the cell periphery along the microtubules [52]. Kinesin-4-based transport of non-cellulosic substances along cortical microtubules is associated with cell wall mechanics in *Arabidopsis thaliana* [13]. The absence of the kinesin-4 family member FRAGILE FIBER1 (FRA1) kinesin results in the accumulation of vesicles around the Golgi apparatus and a reduction in pectin secretion [48]. A recent study found that FRA1 regulates the protein levels of cellulose synthase-microtubules that uncouple protein and microtubule localization, which stabilizes the deposition sites of cellulose and cell wall polysaccharides [49]. In the present study, kinesin-related genes were differentially expressed at the tasseling stage (Table S4). These results suggest that kinesin might be involved in cell wall development through the transport of cell wall non-cellulosic material or influence the deposition of cellulose and cell wall polysaccharides.

Villin is an F-actin regulatory protein with a gelsolin-like core domain and a C-terminal villin headpiece (VHP) [53]. We found that VHP-related genes were differentially expressed between the two SS lines and the NSS line at the ninth leaf and tasseling stages. In the two combinations SS1vsNSS and SS2vsNSS, the expression level of *Zm00001d047453* had the largest differences in cytoskeleton organization (GO: 0007010, *p* = 1.00 × 10^− 2^, log2FC = 1.32–1.41) and microtubule-based process (GO: 0007017, *p* = 1.52 × 10^− 5^, log2FC = 1.60–1.73). Further qRT-PCR assays also indicated that *Zm00001d047453* was differentially expressed between the two SS lines and the NSS line (Fig. 8A). Although the fold change of *Zm00001d047453* was lower in the qRT-PCR assay than indicated by the RNA-seq data, the gene was up-regulated according to both datasets. Bao et al. [54] reported that the deletion of the *VILLIN2* and *VILLIN3* genes in *Arabidopsis thaliana* reduces stem mechanical strength. No defects in secondary cell wall synthesis were detected in that study; however, cortex, interfascicular fiber, xylem, and pith cell numbers significantly decreased, indicating defective sclerenchyma development. Therefore, we speculate that *Zm00001d047453* affects maize stem differentiation and rind development.

## Conclusions

At the tasseling and maturity stages, the two stiff-stalk lines had higher dry weights and hemicellulose, cellulose, and lignin contents per unit length than the non-stiff-stalk line. With regard to microstructure, rind, VBSs, and rind and VBS cell walls were markedly thicker in the two stiff-stalk lines than in the non-stiff-stalk line at the tasseling stage. Transcriptome analysis revealed that these differences might be related to the expression levels of genes involved in cell wall metabolism and the cytoskeleton. Overall, our study has provided new insights into the internode development of maize inbred lines with different stalk strengths.

## Methods

### Materials

Maize inbred lines SJ20104 (NSS), HB08F1 (SS1), and A801 (SS2) were grown at the experimental station (36°90′ N, 117°90′ E) of Shandong Agricultural University. The soil type was brown soil with a sandy loam structure containing 10.5 g kg^− 1^ organic matter, 0.8 g kg^− 1^ total nitrogen (N), 35.2 mg kg^− 1^ readily available phosphorus (P), and 81.8 mg kg^− 1^ readily available potassium (K). N, P, and K fertilizers were applied as described previously [55]. Seeds were sown on 12 June 2019. Each field plot was 4 m × 4.8 m with eight rows (0.60 m between rows, with a planting density of 67,500 plants ha^− 1^).

### Plant height, internode morphology, and RPR measurements

At the maturity stage, plant height was measured from the ground to the top of the tassel. Stem diameter was determined with a vernier caliper arranged perpendicular to the long axis (Fig. 1A) at the center of the third basal internode. The length of the third basal internode was measured with a ruler.

Five individual plants were randomly selected from each maize inbred line at ninth leaf, tasseling, and maturity stages. Days from sowing to ninth leaf, tasseling, and maturity stages were 36, 50, and 102 in NSS; 36, 51, and 104 in SS1; and 38, 59, and 108 in SS2, respectively. NSS, SS1, and SS2 had a total of 16, 16, and 18 leaves at the tasseling stage, respectively. The RPR of the third basal internode was measured with a YYD-1 stalk strength tester (Top Cloud-Agri Technology Co., Zhejiang, China) according to a previous study [56]. In brief, a 1-cm-long probe with a 1-mm^2^ cross-sectional area was inserted perpendicular to the long axis of the cross section at the center point of the third basal internode (Fig. 1A), and the maximum value was recorded as the RPR. Then, the middle part of the third basal internode was cut approximately 2 mm in length (Fig. 1A). The cut part was cut in half along the long axis of the cross-section. One half, designated sample 1, was used for microstructure observation. The other half (as sample 2) was immediately frozen in liquid nitrogen and stored at − 80 °C for RNA extraction.

### Determination of hemicellulose, cellulose, and lignin contents

Another five plants were randomly selected from each maize inbred line for determination of hemicellulose, cellulose, and lignin contents. The entire third basal internode was cut off, heated in an air oven at 105 °C for 30 min, and then dried to a constant weight at 80 °C. The DWUL of the third internode was calculated by dividing the dry weight of the internode by its length. The dried sample was then crushed and passed through a 40-mesh screen. Approximately 1 g of the sifted sample (M1) was placed in a filter bag (with a weight of M0) for measurement of neutral detergent fiber (NDF), acid detergent fiber (ADF), and acid detergent lignin (ADL) contents using an ANKOM 220 fiber analyzer (ANKOM Technology Corp., Fairport, NY, USA) according to a previous study [55]. Hemicellulose, cellulose, and lignin percentages were calculated as follows: hemicellulose (%) = (NDF – ADF)/M1; cellulose (%) = (ADF – ADL)/M1; and lignin (%) = (ADL – M0)/M1. The percentage of each compound was multiplied by DWUL to obtain hemicellulose (HWUL), cellulose (CWUL), and lignin (LWUL) weights per unit length of the third basal internode (mg cm^− 1^). Each component was measured three times per sample.

### Paraffin sectioning and histological staining

Samples for microstructure observation were obtained from the middle part of the third basal internode as described above for sample 1. Tissues at the ninth leaf and tasseling stages were immersed in 50 and 70% formalin–acetic acid–alcohol (FAA) fixative, respectively. Paraffin sectioning procedures were performed as previously described [57]. Samples were dehydrated in a graded ethanol series, soaked in 50% pure ethanol:50% xylene (v/v) for 45 min and 100% (v/v) xylene for 45 min (repeated three times), and then infiltrated with liquefied paraffin at 58 °C. Paraffin sections were produced with an HM360 slicer (Microm, Walldorf, Germany). The sections were dewaxed with xylene for 20 min (repeated twice), anhydrous ethanol for 5 min (repeated twice), and 75% alcohol for 5 min, and rinsed with water. The sections were dyed in Safranin-O for 2 h, and washed in water to remove excess dye. Then, the slices were placed in 50, 70 and 80% gradient alcohol for 3–8 s for decolorization. The slices were dyed in Fast Green solution for 6–20 s and dehydrated with absolute ethanol (repeated three times). Xylene was transparent for 5 min and sealed with neutral gum. Sections were imaged by Nikon DS-Ri2 light microscopy (Nikon, Tokyo, Japan). Measurements of the stem rind, VBS, and cell wall thicknesses were carried out using ImageJ software. Rind thickness was measured 10 times randomly from multiple directions in the cross section of each inbred line (5× magnification). VBS thickness was measured from the thickest part of 10 randomly selected VBSs of each inbred line (10× magnification, shown as a red line in Fig. 2A). Rind cell wall thickness was measured from thick-walled cells in the center of the rind (except for the vascular bundle). VBS cell wall thickness was measured from randomly selected VBSs. Two adjacent cells were measured simultaneously, and 20 pairs of cells were measured (40× magnification). Cell wall thickness was then calculated by dividing each measured value in half.

### RNA-seq analysis

Samples of SS1, SS2, and NSS at the ninth leaf and tasseling stages were used for RNA-seq analysis. These samples for RNA extraction were obtained from the middle part of the third basal internode as described above (as sample 2). Each frozen sample was ground in liquid nitrogen in a 50 mL stainless steel grinding jar with one 20 mm stainless steel ball (AM100, Ant Source Scientific Instrument Co., Beijing, China). RNA isolation and quantification were carried out as described previously [58]. *In* brief, total RNA was extracted from 0.1 g of sample using an RNA extraction kit DP441 (Tiangen, Beijing, China). RNA degradation and contamination were detected by 1% agarose gel electrophoresis, and a NanoPhotometer spectrophotometer (Implen, CA, USA) was used to determine RNA purity. RNA integrity and concentration were assessed using an Agilent 2100 bioanalyzer (Agilent Technologies, CA, USA) and a Qubit RNA assay kit with a Qubit 2.0 fluorometer (Life Technologies, CA, USA), respectively. RNA integrity numbers (RIN) ranged from 7.9 to 9.8 (Table S9), thus indicating that the RNA quality was good.

RNA-seq library construction and sequencing were carried out at Beijing Novogene Bioinformatics Technology Co. (Beijing, China). A NEBNext UltraTM RNA Library Prep Kit for Illumina (NEB, MA, USA) was used for RNA-seq library construction. By using a poly-T oligo-attached magnetic, mRNA was purified from total RNA. First-strand cDNA synthesis was carried out with random hexamer primers and M-MuLV reverse transcriptase (RNase H-). Then, RNase H and DNA polymerase I were used to synthesize second strand cDNA. After adenylation, the AMPure XP system (Beckman Coulter, MA, USA) was used to purify cDNA fragments (250 ~ 300 bp). The libraries were sequenced on an Illumina NovaSeq platform to generate 150 bp paired-end reads. Clean data were obtained by removing adapter-containing reads, reads containing poly-N, and low-quality reads from the raw data. Subsequently, the clean reads were mapped to the maize B73 v4 reference genome (ftp://ftp.ensemblgenomes.org/pub/plants/release-41/fasta/zea_mays/dna/) using HISAT2 [59]. To quantify the gene expression levels, the number of reads mapped to each gene were counted using featureCounts [60], and the expected number of FPKM of each gene were calculated. Expressed genes with FPKM ≥1 were used for the comparative analysis [61]. Differential expression analysis of two samples was performed using the DESeq2 R package (v1.16.1) [62]. The *p-value* were adjusted using the Benjamini and Hochberg algorithm to control the false discovery rate [63], and genes with corrected *p-value* < 0.05 were considered to be DEGs. To understand the functions of significant DEGs, we applied the clusterProfiler R package [64] to enable a GO enrichment analysis. GO terms with a corrected *p-value* < 0.05 were considered to be significantly enriched in DEGs.

### qRT-PCR

Gene-specific primers designed using Primer 6 software were synthesized by Sangon Biotech (Shanghai, China). The primer sequences are listed in Table S10. cDNA was reverse transcribed from total RNA using a PrimeScript RT kit (Takara, Dalian, China) and then analyzed by qRT-PCR on an ABI StepOne Plus Real-Time PCR system (Applied Biosystems, CA, USA). The maize *actin* gene (*Zm00001d010159*) was used as an internal control for normalization of gene expression. The *actin* primer sequences were obtained from a previous study [65]. Each qRT-PCR experiment was repeated three times. Relative expression levels were calculated using the comparative CT (2^−∆∆Ct^) method.

### Statistical analyses

One-way ANOVA and Duncan’s post hoc test were conducted using SPSS 19.0 statistical software (SPSS, Inc., IL, USA).

## Supplementary Information

**Additional file 1: Fig. S1.** Comparison of stem diameters, internode lengths, and plant height.

**Additional file 2: Fig. S2.** Venn diagram of down-regulated DEGs in SS1vsNSS and SS2vsNSS.

**Additional file 3: Fig. S3.** Top 30 significantly enriched Gene Ontology (GO) terms (*p* < 0.05) in DEGs commonly down-regulated in SS1vsNSS and SS2vsNSS at the ninth leaf stage.

**Additional file 4: Fig. S4.** Top 30 significantly enriched Gene Ontology (GO) terms (*p* < 0.05) in DEGs commonly down-regulated in SS1vsNSS and SS2vsNSS at the tasseling stage.

**Additional file 5: Table S1.** Summary of RNA-seq data.

**Additional file 6: Table S2.** List of selected genes related to hydrolase activity (hydrolyzing O-glycosyl compounds) that were up-regulated in SS1vsNSS and SS2vsNSS at the ninth leaf stage.

**Additional file 7: Table S3.** List of selected cytoskeleton organization-related genes that were up-regulated in SS1vsNSS and SS2vsNSS at the ninth leaf stage.

**Additional file 8: Table S4.** List of selected microtubule-based process-related genes that were up-regulated in SS1vsNSS and SS2vsNSS at the tasseling stage.

**Additional file 9: Table S5.** List of selected genes related to transferase activity (transferring hexosyl groups) that were up-regulated in SS1vsNSS and SS2vsNSS at the tasseling stage.

**Additional file 10: Table S6.** Lignin synthesis related genes that were up-regulated in SS1vsNSS and SS2vsNSS at the tasseling stage.

**Additional file 11: Table S7.** List of selected plant-type cell wall organization-related genes that were up-regulated in SS1vsNSS and SS2vsNSS at the tasseling stage.

**Additional file 12: Table S8.** Differentially expressed transcription factors in SS1vsNSS and SS2vsNSS.

**Additional file 13: Table S9.** RNA integrity number (RIN) values.

**Additional file 14: Table S10.** Primer sequences for qRT-PCR analysis.

## Data Availability

The original contributions presented in the study are publicly available. The RNA-seq data have been deposited in the NCBI Sequence Read Archive, accession number: PRJNA752936 (https://www.ncbi.nlm.nih.gov/sra/PRJNA752936).

## Abbreviations

NSS: Non-stiff-stalk
SS: Stiff-stalk
RPR: Rind penetrometer resistance
CesA: Cellulose synthase
CSCs: Cellulose synthase complexes
DWUL: Dry weight per unit length
HWUL: Hemicellulose weight per unit length
CWUL: Cellulose weight per unit length
LWUL: Lignin weight per unit length
VBS: Vascular bundle sheath
FPKM: Fragments per kilobase of transcript sequence per million base pairs sequenced
DEGs: Differentially expressed genes
GO: Gene Ontology
XET: Xyloglucan endo-transglycosylase
GH: Glycosyl hydrolase
GT: Glycosyltransferase
XTH: Xyloglucan endotransglucosylase/hydrolase
PAL: Phenylalnine ammonialyase
4CL: 4-coumarate:CoA ligase
TF: Transcription factor
XET: Xyloglucan endotransglycosylase
IRX: Irregular xylem
Arp2/3: Actin-related protein 2/3
FRA1: FRAGILE FIBER1
VHP: Villin headpiece
NDF: Neutral detergent fiber
ADF: Acid detergent fiber
ADL: Acid detergent lignin
