# Supplementary material for: Transcriptome analysis reveals the mechanism of internode development affecting maize stalk strength
Source: BMC Plant Biol. 2022 Jan 24;22:49. doi: 10.1186/s12870-022-03435-w (PMC8785456; doi:10.1186/s12870-022-03435-w)
Supplement: Supplementary file 1 — Additional file 1: Fig. S1. Comparison of stem diameters, internode lengths, and plant height. [file 12870_2022_3435_MOESM1_ESM.docx]

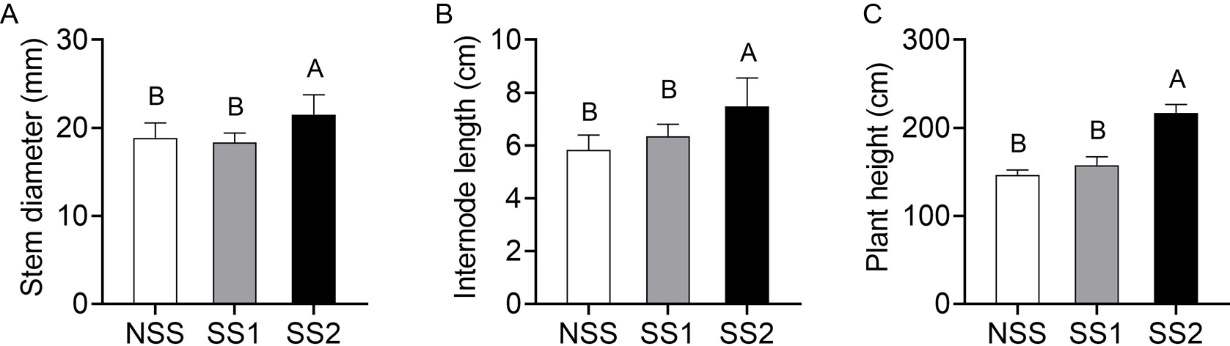


**Fig. S1** Comparison of stem diameters, internode lengths, and plant height. At the maturity stage, the stem diameter (A) and internode length (B) of the third basal internode and plant height (C) were measured. Error bars represent standard deviation (n = 10). Different letters indicate significant differences at the same developmental stage (*p* < 0.01 by one-way ANOVA). SS1: stiff-stalk-line HB08F1; SS2: stiff-stalk-line A801; NSS: non-stiff-stalk-line SJ20104.
