# Supplementary material for: Transcriptome analysis reveals the mechanism of internode development affecting maize stalk strength
Source: BMC Plant Biol. 2022 Jan 24;22:49. doi: 10.1186/s12870-022-03435-w (PMC8785456; doi:10.1186/s12870-022-03435-w)
Supplement: Supplementary file 2 — Additional file 2: Fig. S2. Venn diagram of down-regulated DEGs in SS1vsNSS and SS2vsNSS. [file 12870_2022_3435_MOESM2_ESM.docx]

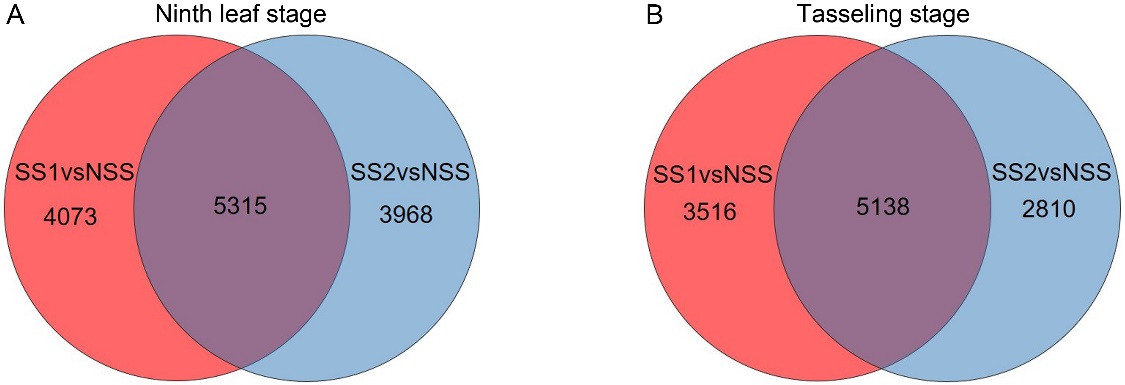


**Fig. S2** Venn diagram of down-regulated DEGs in SS1vsNSS and SS2vsNSS. (A) Ninth leaf stage. (B) Tasseling stage. SS1vsNSS: stiff-stalk-line HB08F1 compared with non-stiff-stalk-line SJ20104; SS2vsNSS: stiff-stalk-line A801 compared with non-stiff-stalk-line SJ20104.
